# Tracking the Development of Community Engagement Over Time: Realist Qualitative Study

**DOI:** 10.2196/47500

**Published:** 2024-05-15

**Authors:** Esther de Weger, Hanneke Drewes, Katrien Luijkx, Caroline Baan

**Affiliations:** 1 Department of Tranzo, Tilburg School of Social and Behavioural Sciences Tilburg University Tilburg Netherlands; 2 Vrije Universiteit Amsterdam Amsterdam Netherlands; 3 Siza Arnhem Netherlands

**Keywords:** community engagement, citizen involvement, health care, decentralization, realist evaluation

## Abstract

**Background:**

A growing interest in engaging communities in the development of health care services and communities has not automatically led to progress or consensus as to how to engage communities successfully, despite the evidence base showing how to leverage enablers and alleviate barriers.

**Objective:**

To bridge the gap between the evidence base and which community engagement (CE) approaches have actually been applied in practice over time, this study aims to investigate how CE approaches have changed over the past 4 years in 6 different regions in the Netherlands and citizens’ and professionals’ experiences underlying these changes.

**Methods:**

For the last stage of a multiple case study following the development of CE approaches in 6 different regions in the Netherlands, a realist qualitative case study was conducted. To investigate how CE approaches had changed over the past 4 years, data from the entire 4 years of the study were used, including documents, interview transcripts, and observations. To examine citizens’ and professionals’ experiences underlying these changes, new interviews were conducted. The latest interview results were discussed with a panel to ensure the results had face validity.

**Results:**

The regions had implemented different types of CE approaches over the past 4 years and were adapting these approaches over time. Many of the (remaining) approaches may be operating on a smaller scale. The study identified the following overarching themes along which CE had been adapted: fewer region-wide approaches and more community-focused approaches, more focus on building relationships with (already engaged) citizens and community-led initiatives, and more focus on practical and tangible health promotion and social cohesion activities and less focus on complex “abstract” programs. The study identified a further 4 overarching themes highlighting citizens’ and professionals’ experiences underlying these changes in the CE approaches: a lack of engagement environment, need for facilitative leadership from organizations, need for a clear and shared vision underscoring the importance of CE, and misalignment between citizens’ and professionals’ perspectives and motivations for CE. All participants had experienced the engagement environment as insufficient. To support CE, professionals experienced the need to develop and receive more facilitative leadership and to develop approaches better equipped to involve citizens in the decision-making process. Citizens experienced the need to better align citizens’ and professionals’ motivations and aims for CE approaches and to receive longer-term financial support for their community-led initiatives.

**Conclusions:**

This study suggests that CE has not yet been embedded within organizational cultures. This has arguably meant that the (remaining) CE approaches are operating on a smaller scale. To enable the further development of CE approaches, an investment in the engagement environment and a shared vision is required. Only then could CE within the regions move beyond the more seemingly smaller-scale CE approaches.

## Introduction

### Background

Over the past few decades, public sector organizations have increasingly been trying to engage citizens in shaping and improving health care services, neighborhoods, and healthy living environments [1,2]. The idea behind community engagement (CE) is that through citizens’ involvement services and policies will better reflect communities’ experiences and better address their needs [3-7]. The aim of CE approaches is to involve citizens in the decision-making, planning, designing, governance, or delivery of services and policies. CE approaches can range from consultation where citizens have limited power to influence decision-making to partnership and (shared) leadership, where citizens have decision-making control [1,8,9]. The approaches can take many different forms, including citizen advisory panels, citizen budgetary forums, peer service delivery, and community-led initiatives [1].

However, as Beresford [10] noted, this increasing and widespread interest in involving communities in the development and improvement of health care services and living environments does not automatically mean progress or consensus as to how to do so meaningfully and successfully for either organizations or communities. Previous literature hints at the barriers that have hindered this progress toward CE over the past few years. For example, Cook and Kothari [11] argued that many participatory processes are often undertaken uncritically based on the perceived wisdom of the overwhelming benefits of CE. However, these formulaic approaches often impose the views, objectives, and aims of organizations onto communities, resulting in benefits that primarily serve the organizations themselves, or, more commonly, fail to deliver benefits to either organizations or communities. Previous literature has described other important factors driving this lack of progress, such as power imbalances between organizations and communities, engaged citizens’ limited credibility according to professionals, communities’ lack of influence in decision-making processes, misaligned interests between organizations and between organizations and communities, or a lack of a clear and shared vision for CE [5,10,12-14].

Despite the wealth of previous literature identifying important barriers and enablers to the progress of CE, health and care organizations are still searching for how to implement their own successful CE approaches and largely have not yet taken the required steps to leverage these identified enablers [14] or improved the engagement environment sufficiently [14]. Moreover, previous literature has not investigated how CE has developed over time. Because of this gap between the evidence base and how the implementation process of CE approaches over time is actually experienced in practice, this study examined how CE developed during the course of 4 years in practice. To provide insight into the development of CE in the Netherlands, we conducted a 4-year multiple case study investigating how 6 different regions are developing and implementing their own CE approaches. The initial phase of the study involved conducting an international rapid realist review to identify the barriers and enablers for engaging communities. This review resulted in the development of 8 guiding principles for the successful implementation of CE [13]. Subsequently, these principles were tested in practice through various case studies, leading to the identification of a ninth guiding principle [13-16].

### Objective

Building on the previous stages by using the guiding principles as program theories, this paper describes the final stage of the study. This final case study aims to investigate how CE has changed over the past 4 years in the 6 regions and to examine citizens’ and professionals’ experiences underlying these changes. This paper explored the following research questions:

1. What CE approaches have been applied, and how have these approaches changed over the past 4 years?
2. What are citizens’ and professionals’ experiences underlying the changes in CE approaches? What are the contextual factors and mechanisms explaining these experiences?

## Methods

### Overview

This paper presents the last stage of this multiple case study (T4). This final stage examined how CE approaches have changed over the past 4 years and what citizens’ and professionals’ experiences were underlying these changes. The study was informed by the realist evaluation (RE) approach. The RE approach seeks to explain the causal relationship between contexts, mechanisms, and outcomes in particular programs of interest [17]. In this way, the study sought to understand the causation behind the changes in CE approaches and to understand which (enabling and constraining) mechanisms were triggered within the (changing) contexts of the 6 regions and how these influenced citizens’ and professionals’ experiences of developing CE (Textbox 1 [13], [14], [18]; Multimedia Appendix 1).

**Community engagement–oriented definitions of realist concepts [13,14,18].**

**Intervention**

This refers to interventions’ implemented activities, strategies, and resources [19], for example, citizen advisory panel meetings or neighborhood organized workshops.

**Context**

This pertains to the backdrop of an intervention and includes the preexisting organizational structures, cultural norm of the community, the nature and scope of preexisting networks, and geographic location effects [20-22].

**Mechanism**

This refers to what “triggers” participants to want to participate or not in an intervention. “Mechanism” does not refer to the intentional resources offered or strategies implemented within an intervention. Mechanisms usually relate to cognitive, emotional, and behavioral responses to intervention resources or strategies [20]. Mechanisms are usually hidden, sensitive to variations in context, and generate outcomes [23], for example, citizens feeling more empowered because of learning opportunities.

**Outcome**

Refers to intended, unintended, or expected intervention outcomes [20], for example, sustainability, quality, and integration of services (macro); citizens’ level of involvement in health and care services (eg, in designing policies; meso); and citizens’ health and well-being outcomes (micro).

**Context-mechanism-outcome (CMO)**

To understand how certain contextual factors shape or trigger the mechanism, causal links are expressed through “context-mechanism-outcome configurations.” Formulating and refining CMOs is largely how researchers analyze data in realist evaluation as it allows a deeper understanding of which (aspects of) interventions work, for whom, under which circumstances, and to what extent [24]. CMOs are also used to generate or refine program theories, which in turn help shape the final product of an evaluation (eg, recommendations). CMOs are also used to generate or refine program theories.

For this 4-year multiple case study, 6 different regions within the Netherlands were chosen as the research sites (Multimedia Appendix 2). The different contextual factors and the different CE approaches within the regions helped to compare and contrast citizens’ and professionals’ experiences accordingly. For the first research question regarding what CE approaches have been applied and how the approaches have changed over the past 4 years, data from the entire 4-year multiple case study were used. Data sources to answer the first research question included (strategy) documents, completed observation templates (based on stakeholders’ meetings, workshops, and activities), transcripts of (group) interviews with stakeholders, and reference panel workshop discussions [13-16]. For the second research question, which focuses on understanding the experiences of both citizens and professionals that underlie changes in CE approaches, only the most recent round of data collection (T4) was used. Data sources for this last data collection round included (new strategy) documents, (new) interviews with stakeholders, and the last reference panel workshop (T4).

### Recruitment and Study Sample

The last data collection round was first based on interviews with professionals (n=7; this included policy makers, project managers, local councilors, and health care professionals) and with citizens involved in organizationally led projects and community-led initiatives (3) in the 6 different regions in the Netherlands (T4; Table 1; Multimedia Appendix 2). For this study, purposive sampling [25] was used to ensure different professionals and citizens from each of the 6 regions were included in the sample. As much as possible, the same citizen and professional participants as in the previous stages of the 4-year multiple case study were approached and interviewed, thus hoping to enable a better view of how participants’ experiences had developed over the years. Professionals and citizens were recruited through the reference panel members’ networks. Almost all approached participants agreed to take part in video or telephone interviews and had signed consent forms, except for participants in region B who only agreed to take part in the reference panel. Ultimately, a total of 10 interviews (1 dyad with a local councilor and a project manager), each lasting approximately 1.5 hours were conducted. Unfortunately owing to the COVID-19 pandemic, researchers were prevented from meeting participants face-to-face and interviews could only be conducted remotely through video or telephone calls. Interviews were conducted until the authors agreed the point of data saturation was reached or when no new themes emerged and when there was a high rate of recurrence of responses [26].

**Table 1:** Description of the regions and sample size [14].

Region	Region description	Sample size at this stage of the study (T4)
A	Rural region made up of several smaller municipalities, struggling with aging population and economic decline (number of residents=106,500) Expected average age at birth and expected average age in good health were 80.9-81.6 years and 47.9 years Socioeconomic status variable according to different neighborhoods with pockets of deprivation and more affluent areas Quality of life in neighborhoods varied accordingly. Region with declining and aging population	1 interview with policy maker 1 interview with engaged citizen 1 (different) policy maker involved in the reference panel
B	Region with a mix of rural and urban areas, with significant health disparities (number of residents=270,000) Expected age at birth and expected average age in good health 78.2 to 79.6 years and 45.2 years Socioeconomic status variable according to different neighborhoods but included more deprived neighborhoods due to the traditional industry in the area having been closed down Quality of life varied accordingly	2 patient and public involvement professionals engaged in reference panel (not interview)
C	Rural municipality with 13 different villages with favorable unemployment and welfare support rates compared to the national average (number of residents=27,500). Expected average age at birth and expected age in good health 82.0 to 48.7 years Socioeconomic status higher than the national average Quality of life higher than the national average	2 interviews with 2 policy makers
D	Region with a mix of rural and urban areas, with significant health disparities and less favorable unemployment and welfare support rates compared to the national average (number of residents=27.500) Expected average age at birth and expected average age in good health 80.5 to 84.7 years and 45.2 to 47.1 years Socioeconomic status variable according to different neighborhoods but includes more deprived neighborhoods due to the traditional industry in the area having been closed down Quality of life varied accordingly but has an aging population	1 interview with a professional 2 citizens engaged in the reference panel
E	Rural region made up of 4 municipalities with pockets of health disparities (number of residents=120,000). Expected average age at birth and expected average age in good health 80.4 to 82.0 years and 48.2 years Socioeconomic status higher than the national average but with pockets of significant deprivation (differences between the statuses) Quality of life on average higher than the national average	1 dyad interview with 1 policy maker and 1 project manager
F	Suburban municipality with favorable unemployment and welfare support rates compared to the national average (number of residents=41,000) Expected average age at birth and expected average age in good health 81.3 years and 45.5 years Socioeconomic status in line with national average Quality of life in line with national average	1 interview with a public health professional 1 interview with an engaged citizen 1 interview with an engaged citizen Same citizens engaged in the reference panel

### Reference Panel

The 4-year multiple case study was conducted in collaboration with a reference panel. The panel consisted of stakeholders involved in developing CE approaches within the 6 different regions, including policy makers; involved citizens; members of patient and public involvement organizations; and experts in the field of public health, health inequalities, and citizen participation. The panel, therefore, helped to ensure that the study addressed stakeholders’ questions regarding CE and addressed relevant gaps in the literature. For this data collection round (T4), the panel also helped with the sample selection and recruitment process. Furthermore, the interview findings were discussed with the reference panel to further enrich the results and to ensure that the results had face validity. Multimedia Appendix 2 highlights the participants (n=17) present during the workshop to whom the interview findings of this last study were presented.

For the final study (T4), participants were asked to draw up their own storyboards to reflect on the development of their own CE approaches during web-based or telephone interviews. Owing to the COVID-19 pandemic and to reduce the burden on participants, participants were given the option of drawing or writing on the web-based storyboard themselves or instructing the researchers how to do it for them. The storyboards aimed to enable participants to reflect in a more participative and creative way on their experiences and perceptions regarding the development of CE more broadly. The storyboards highlighted not only the broader experiences but also specifically the enablers and barriers and the support needs going forward [27-29]. Participants were asked to consider the following three questions when drawing up their storyboards: (1) which successful steps they had taken with the development of CE and which enablers they had experienced, (2) which negative results they perceived during the development of CE and barriers they experienced, (3) how these enablers and barriers have changed their CE approaches going forward.

During the second part of the interview, participants were asked to consider their storyboard and imagine they had to advise someone else to successfully develop a CE project. They were asked to note their advice down on notecards. After this, participants were asked to cluster their advice into two groups: (1) what advice they already follow themselves and (2) what advice they struggle to implement themselves. By clustering current enablers and barriers in this way, and discussing the underlying reasons, the study aimed to highlight practical advice to enable stakeholders to implement their new CE approaches [27,28,30]. The interview data were collected between February and May 2020.

After the initial analysis of the interviews and the secondary data, the anonymized results were shared and discussed during a workshop with the reference panel (Multimedia Appendix 2). This further refined and enriched the results. The workshop was held in January 2021.

Finally, to supplement and triangulate the interview data, the authors conducted a document analysis from the field notes taken over the 4 years of study and from the regions’ strategy documents.

### Data Analysis

To help answer the first research question (regarding the changes in CE approaches), the authors re-examined previous interview transcripts, observation templates, and documents. The authors also classified the CE approaches at “the consultation level”—whereby citizens provide information to organizations, “the communication level”—whereby citizens receive information from organizations, or “the participation level”—whereby citizens are actively engaged in dialogue with organizations and are actively involved in the planning, implementation, or decision-making—of approaches as in line with the findings of the previous studies [14]. To answer the second research question (regarding citizens’ and professionals’ underlying experiences), the same researchers who had been involved during the entire 4 years of this study applied an inductive and deductive analysis approach to the last round of interviews (T4). Inductively, we searched for (1) changes in CE approaches; (2) citizens’ and professionals’ experiences in developing and implementing CE, including enablers and barriers; and (3) required support to further develop CE. After this open coding and analysis, the researchers also deductively applied the guiding principles within the coding structure and analysis approach. These guiding principles are as follows: (1) ensure staff provide supportive and facilitative leadership to citizens; (2) foster a safe and trusting environment enabling citizens to provide input; (3) ensure citizens’ early involvement; (4) share decision-making and governance control with citizens; (5) acknowledge and address citizens’ experiences of power imbalances between citizens and professionals; (6) invest in citizens who feel they lack the skills and confidence to engage; (7) create quick and tangible wins; (8) consider both citizens’ and organizations’ motivations; and (9) develop a shared vision with clear roles for professionals and citizens, ensuring communities’ diversity is reflected within the vision [13,14].

To examine how CE has been developed and changed over the past 4 years and what citizens’ and professionals’ experiences were underlying these changes in 6 different regions in the Netherlands, the authors constructed context-mechanism-outcome (CMO) configurations within each interview transcript to examine the contextual factors and mechanisms underlying these changes and to investigate participants’ experiences. Interviews were thus coded and analyzed using CMOs, which were drafted and analyzed in MAXQDA (VERBI GmbH) by EdW, and discussed by all authors. To aid authors during the data analysis process and to ensure consistency and transparency, the authors applied the same CE-oriented definitions of “interventions,” “contexts,” “mechanisms,” and “outcomes” (Textbox 1). The clustering followed a sequential and iterative process that has been applied in previous studies and described elsewhere [13,14]. CMOs were coded and clustered into (1) changes in CE approaches over the past 4 years, (2) participants’ experiences (including enablers and barriers), and (3) required support to further develop and implement CE. The authors discussed the clusters and thematically analyzed, reviewed, and discussed them again. The final draft of the clustered CMOs was shared with all authors to confirm and refine the themes (Multimedia Appendix 3). Afterward, for the deductive analysis, the transcripts and the CMOs were coded and clustered according to the 9 guiding principles.

### Ethical Considerations

The study received ethics approval from Tilburg University (reference EC-2017.96). All participants were provided with information letters concerning the study and had time to ask any questions they may have had. It was also made clear that participation was completely voluntary. Afterward, all participants signed forms stating their consent to participate. This is in accordance with Dutch national guidelines.

## Results

### Overview

The following section will first describe how CE approaches have changed over the past 4 years (Table 2). The study indicates that there are 3 overarching themes regarding the changes in CE approaches. Theme 1: moving away from region-wide approaches to more community-focused approaches. Theme 2: more focus on building relationships with (already-engaged) citizens and community-led initiatives. Theme 3: more focus on practical and tangible health promotion and social cohesion activities instead of on more complex “abstract” programs

**Table 2:** Cross-sectional summary of community engagement approaches over the past 4 years [14].

Region	Interview round 1: 2016-2017	Interview round 2: 2018-2019	Final interview round: 2020-2021
Region A	Communication level Regional web-based community platform highlighting the projects and meetings being organized by the regional health care board. As part of the web-based community, hoped to develop “an instrument” to increase the region’s self-management capacity (not developed). Consultation level Regional web-based community platform to create propositions and test these among citizens and health and care organizations. In this way, the regional health and care board hoped to learn key issues facing health and care organizations and the residents (eg, experiences, needs, projects, or meetings). Participation level Regional web-based community platform, supported by occasional physical meetings, to enable dialogue between residents, municipalities, health care professionals, clients, schools, and volunteer clubs (eg, sports clubs), businesses, and health and care organizations about how health and care services can become futureproof and maintain its quality and efficiency. Online community aimed at increasing social between engaged or interested residents, organizations, and other stakeholders of the regional health and care system.	Consultation level Regional public health organization, commissioned on behalf of one of the region’s municipalities, conducted interviews and focus groups with residents to discuss their perceptions and experiences of what it is like to live in that municipality (completed). Public health organization region A held informal dinner events with older residents to discuss their current and future health and care needs and the sort of local amenities they would like to have available in the municipality (completed). Regional living room: supports organizations and residents to address urgent health and care issues in the region. “Living rooms” across the province have been set up for residents to investigate such issues together (ongoing but by different organization). Participation level Regional web-based community: online platform, which enabled all residents and professionals within the region to share and collect information regarding the region’s health care system. The platform also enabled residents to share their ideas of how future health and care services should take shape in the region (disbanded).	Participation level Municipality within the region had started a project to improve the living environment of 1 village with the aim of also contributing to the green energy transition in the municipality. The municipality together with the village council had set up the project (completed). Municipality searching for ways to include citizens (especially older citizens) in the reconfiguration of health and care services within 1 municipality. To date, it had not found a way to involve citizens on the “participation level” (ongoing).
Region B	Communication level Developed guidelines or how-to guide to stimulate the engagement of the >65 years age group for specific neighborhood projects and development. Guideline was developed through interviews with residents aged >65 years in the region about their engagement experiences (completed). Participation level Looking for ways to leverage all the separate existing citizen representative bodies (eg, village councils, client councils, and church councils) that can be leveraged to increase citizen representation on the regional governance level. Currently, these approaches all operate separately from each other and on a more local level. A retired surgeon and a representative of a regional PPIa organization were members of the regional health care governance board (surgeon no longer involved).	Communication level PPI^a^ organization freely distributed a magazine to all residents in the region and promoted healthy living and community engagement activities and projects (ongoing). Participation level “WeHelpen” web-based platform that enables residents to ask for and provide each other with informal help, from mowing each other’s grass to social visits and doing groceries for the less abled (ongoing). A local resident and a representative of a regional PPI organization were members of the regional health care governance board (resident no longer taking part, PPI representative still present).	Communication level PPI organization “educated” citizens on self-management and the concept of positive health, for example, workshops and conferences (ongoing). Participation level PPI organization advised health and care organizations on how to involve patients and citizens in their projects (ongoing). PPI organization provided training to patients on how to be involved (ongoing).
Region C	Communication level Using visualizations of broader health and care concept “positive health” to discuss and develop municipal-wide policies and projects with residents and using the visualization as a financial lever for change (only projects highlighting they contribute to the positive health of residents; ongoing). Looking to develop jargon-free language to engage residents (ongoing). Participation level Looking for ways to engage children, young adults, and parents to help develop municipality’s youth care policies (ongoing).	Participation level Municipality professionals working to establish closer working relationships with residents, local sports clubs, and village council (ongoing). Municipality was working to establish closer relationships with schools, parents, and students to engage them in the development and improvement of the municipality’s youth policy (ongoing). Involved citizens in the development of integrated local health policy (completed).	Consultation level Used interviews to gain insight into low-income residents’ experiences and needs regarding low-income support and thus to align low-income policies more to low-income residents’ needs (completed).
Region D	Communication level Looking for “tools” to increase citizens’ awareness regarding positive health and to engage citizens in projects regarding positive health (completed). Took part in health care markets to raise awareness for healthy living lifestyles (completed). Participation level Started their own nonmandatory client council with the idea that clients within the region can be involved in creating new projects and to share which aspects are important to their own positive health (disbanded). Considering developing their own “Digipanel” to enable citizens to share their thoughts on policy developments (not developed).	Consultation level Conducted patient satisfaction surveys for general practices as part of a new quality improvement system whereby practices will be monitored as to whether they are implementing measures to improve areas highlighted in the survey (in an attempt to make general practices more accountable to the patients; completed and considering running again). Community-led initiative kicked-off with passing around a “village diary.” The volunteers went door-to-door with the diary to ask their neighbors to write something about their village, for example, what they liked about the village and what local amenities they felt were missing. Volunteers then used the diary as the foundation for the community-led initiative (completed). Communication level Workshops for residents with the aim of promoting “positive health” (ongoing). With the aim of setting up better working relationships between a local municipality and the community-led initiatives, a PPI and citizen representative organization held separate workshops with the municipality and with the initiatives to gain insight into how to improve their collaboration. At the end of the learning program, the organization was hoping to have 1 joined workshop (completed). Participation level Primary care group’s client council (disbanded). A community-led village initiative was set up when the village’s only general practitioner retired. The community-led initiative, had at the time of interviewing, set up a multidisciplinary medical center, a free library and reading nook, a shared neighborhood-allotment, social activities and evenings, and were working to expand the center’s remit. Resident village support worker who maintained close links within their own communities and ensured that the health, care, and living needs of their neighbors were being addressed (whenever possible by village residents themselves and otherwise, the village support worker ensured appropriate support from the municipality was made available; ongoing).	Participation level Community-led initiative continued to grow and looked to keep promoting social cohesion and social activities. They especially looked to keep this going during the COVID-19 crisis. Also looking to take on a commissioning role for certain health and care services. Resident village support worker continued his linking pin role, especially during the COVID-19 crisis.
Region E	Communication level Several municipalities had conducted a “health scan” with residents to investigate and discuss what key issues they were facing (completed). Participation level The biggest insurance companies, local municipalities, and health and care providers had set up a Policyholder Cooperation to ensure policyholders could have a say in which services should be included within the insurance package and could help shape the local health care system. They wanted to provide all policyholders to be able to vote on important decisions and were looking to recruit policyholders to be on the board. Residents within some of the villages had created some groups to raise awareness for healthy living lifestyles (eg, through walking groups, setting up social meetings, and running events). Municipality is looking for ways to support these groups (ongoing).	Communication level Annual policyholder events and workshops promoting positive health (disbanded) Local municipalities were establishing closer relationships with community-led initiatives and sports clubs with the aim of improving children’s and young people’s health (ongoing) A “Self-care for me” website, which enabled local residents to score their own health. The local municipalities were hoping to get local businesses involved to set up “fun challenges” improving residents’ health (ongoing). Participation level The biggest insurance companies, local municipalities, and health and care providers had set up a Policyholder Cooperation to ensure policyholders could have a say in which services should be included within the insurance package and could help shape the local health care system (disbanded).	Communication level Looking to implement 1 contact person at municipalities for community-led initiatives.
Region F	Participation level Project initiated by regional public health organization to support low-income families. Parents from these families are involved in the projects highlighting important priorities and activities. Parents are also involved in the implementation of activities (completed). Community-led initiative set up to promote the positive health in the community by organizing health promotion activities (eg, benches along walking paths; ongoing).	Participation level Community-led initiative that designs and implements health promotion projects, activities, and workshops (eg, implementing benches along walking paths, workshops regarding positive health, and developing health promotion apps; nearly disbanded, but continued).	Participation level Community-led initiative continued but with different citizens involved at the governance level. In addition, the community-led initiative was also being supported by a public health professional (ongoing).

^a^PPI: patient and public involvement.

Following on, the paper will also examine participants’ underlying experiences throughout the CE process (including enabling and constraining experiences and support needs to further develop CE). The study indicated another 4 overarching themes related to these experiences:

- Theme 4: lack of investment in the engagement environment
- Theme 5: need for facilitative leadership
- Theme 6: need for a clear and shared vision underscoring the importance of CE
- Theme 7: misalignment between citizens’ and professionals’ perspectives and motivations for CE

Throughout this section, examples of CMOs will underpin the results, and further CMO examples can be found in Multimedia Appendix 3.

### Changes in Applied CE Approaches

#### Overview

Within all 6 regions, there had been changes within both the organizationally led CE projects and community-led initiatives. Table 2 shows a summary of CE approaches that have been implemented over the past 4 years within the 6 regions to improve communities’ health and well-being and to improve the health and care systems. This summary is not an exhaustive list, and the final column is focused on newly implemented CE approaches compared to previous years. Table 2 highlights that after 4 years, most approaches and underlying activities could still be classified at the “consultation,” or “communication” level and that some “participation level” approaches within the regions had been disbanded (ie, the web-based community platform in region A, the client council in region D, and the policyholder cooperation in region E). Furthermore, although this list is not exhaustive, the results as shown in Table 2 seem to underscore that the implementation of CE in the regions is in development and that most CE initiatives are now small scale. Though some of the regions were trying to address this, for example, the patient and public involvement organization in region B had been trying to embed CE within organizational cultures through training, and the organization in region A had been trying to build relationships with engaged citizens.

#### Theme 1: From Regional Focus to Community Focus

Table 2 also highlights that the 6 regions have adapted their CE approaches over the past 4 years. First, some regions had shifted their CE approaches from having a more regional focus to a community-based focus. For example, the policyholder cooperation in region E had been disbanded as its focus on complicated, regional issues such as the regional economy and the viability of the hospital was seen as too far removed from “average” citizens’ lived experiences. That is why, at the time of interviewing, the regional board was looking for ways to take a more community-focused approach by involving and facilitating citizens in practical health promotion activities aimed at improving the health and social cohesion of communities, thus hoping to connect more with the lived experiences of citizens and communities.

#### Theme 2: Building Relationships With (Already Engaged) Citizens

Second, and likely relatedly, some regions were trying to change their CE approaches to focus more on building relationships with communities and engaged citizens. For example, policy makers in region A have noticed a slow shift in mindset within municipalities. Where originally municipalities thought they knew what was best for communities, policy makers (through positive experiences of involving citizens in developing and renewing social spaces) are seeing the benefit of building relationships with (engaged) citizens and communities and involving citizens in the design phases of projects, instead of presenting finalized plans to citizens.

#### Theme 3: Shift to More Practical and Tangible Projects

Third, and again likely relatedly, most of the regions have started focusing more on practical, tangible CE projects with activities aimed at improving the health and social cohesion of communities (eg, placing benches in parks to encourage older residents to go for walks, walking groups, and living library events; Table 2). For example, the citizens within the community-led initiative in region F had organized many smaller-scale practical projects and events as the tangible aspects of health promotion and social cohesion activities were seen as more motivating than, for example, the development of a web-based app for individual use:

It’s not for nothing that things [CE] start in the villages...It’s got to do with the small scale that makes people want to self-organise and maybe it helps with the collaboration, it’s]always easier with knowing people and after that maybe there’s the right energy whereby people want to do stuff [get engaged/self-organise]. So that smaller scale, always has something to do with it.

Region F, policy maker, male

### Citizens’ and Professionals’ Experiences

Underlying the nature of changes in the CE approaches, as described in the previous section, were citizens’ and professionals’ experiences (Multimedia Appendix 3). Overall, citizens and professionals had experienced many of the previous approaches as too far removed from citizens’ lived experiences to be successful and felt that further improvements were necessary to further develop CE.

#### Theme 4: Lack of Engagement Environment

First, and most prominently, both citizens and professionals had experienced a lack of investment in, and a need to improve, the engagement environment. This lack of investment prevented CE from being fully embedded within organizational cultures. Both citizens and professionals experienced the need for further investments, that is, in the form of resources and funding for activities and initiatives, staff with CE skills and know-how, and space and time to build relationships with a wider range of citizens and to innovate CE approaches (Multimedia Appendix 3). The study indicated that participants experienced the need for 2 different types of investment. The first type was a “softer,” more cultural type of investment. For example, in regions A and C, the organizational culture used to be that the municipalities decided everything, but because of laws such as the Participation Act (2015) and the Living Environment Act (2021), they have been forced to review the role citizens have (context). Furthermore, the newer generation of policy makers has been trained to see the value of CE and has experienced the positives of involving citizens in projects and policy making (context). Because of this, policy makers are increasingly seeing and believing the value of CE and at the same time experiencing that this belief is not supported by the wider municipality or their management (mechanism). They felt this slows down the cultural change required within organizations to enable successful CE approaches (outcome). At the same time, participants also described the more “tangible” types of investments required to enable the further development of CE approaches. For example, the community-led initiative in region F was able to organize health promotion and social cohesion activities successfully, despite the fact that organizations had not provided long-term financial support (context) and despite a drop in the number of volunteers (context). The volunteers experienced the organization of such activities as draining without support as it cost them a lot of time and energy (mechanism). This made it difficult for the community-led initiative to ensure they could keep organizing such activities in the long term (outcome). While one of the organizations in region D highlighted the need to develop CE skills and know-how. For example, one of the organizations had applied for a subsidy to involve organizations from the cultural or creative sector to develop new and innovative ways to involve citizens within the Positive Health Network (context). Because when health and care organizations think about CE, they end up involving citizens in the traditional (more limited) way (mechanism). Unfortunately, the subsidy was rejected, which meant that the search for new innovative ways to involve citizens remains (outcome):

I think I’ve been lucky in certain ways, that our conservative local councillor left and a new councillor took his place. And that new councillor said to me: “why don’t you just try something.” If I’d still had a councillor who kept saying: “no, that’s not how we do it.” Then I wouldn’t have had the space to involve the citizens like that.

Region A, policy maker, male

My story, what are the blockades? I see that in the community and for the community-led initiative a lot of balloons [projects] are raised. Sometimes with a small pot of money. But when that pot of money is emptied, the balloons are popped. There’s too little space for embedding things.

Region F, citizen, female

#### Theme 5: Need for Facilitative Leadership From Organizations

Second, and relatedly, both citizens and professionals had experienced a lack of, and need to provide and receive, facilitative leadership. Furthermore, both citizens and professionals were also trying to develop new leadership. For example, the community-led initiative in region F was launched 5 years ago with a local health care professional in the lead but without a clear governance or leadership structure (context). Initially, 4 board members were selected but most were health care professionals within the community as well (and were thought to have vested interests removed from “regular citizens”; context). Citizens felt these members were unapproachable and the health care professional who had launched the initiative was not motivated to take up the leadership role in the long term (mechanism). This lack of clear leadership made it difficult for the engaged citizens to know what the decision-making process was or who to turn to with their project ideas (outcome). That is why when the members of the old governance board left, the new members (all citizens) decided to be approachable and discuss and align everyone’s goals clearly. An example of how professionals were experiencing CE was expressed by policy makers in region C. The negative experiences of involving citizens when the municipality had already developed the plan meant they started searching for a new approach to CE (context). The new approach is based on sharing the problem and issues the municipality is trying to address with communities with the aim of improving the collaboration between engaged citizens and organizations (context). Sharing the problem fosters commitment among engaged citizens and organizations, motivating them to consider potential solutions (mechanism). Through this new, more facilitative approach, everyone (municipality, engaged citizens, and organizations) has gained more understanding of each other (outcome):

I think you need leadership and guts, you have to be able to reach out to citizens and to show that you can let go [of control]. Several of our administrators find that difficult. They’re used to being in charge and in control. But actually, here we say “don’t be in change or in control, but ask questions. Create and connect. That’s a totally different way of providing leadership.”

Region E, local councilor, male

I think that you just have to talk to each other, what you want from the initiative, as professional and as volunteer. You have to create the atmosphere where such things can be talked about, and both sides have to listen...that requires that you make yourself vulnerable thus open to the ideas, suggestions and comments of others.

Region F, citizen, male

#### Theme 6: Need for a Clear and Shared Vision Underscoring the Importance of CE

Third, both citizens and professionals continued to seek and emphasize the need for the implementation of a clear and shared vision underscoring the importance of CE. Policy makers in region A highlighted that old habits of policy makers of not sharing control with citizens die hard, especially as there is not a clear or shared vision for the relevance of CE within the municipality (context). The lack of shared vision has prevented policy makers from experiencing and seeing CE as part of their “day-to-day” business (mechanism). That is why the required culture change to embed CE activities within organizations and on a regional level successfully has taken a long time (outcome). Some policy makers speculated that this lack of CE vision is because municipalities only involve citizens (through the bare minimum effort) because national policies such as the Participation Act (2015) have dictated they do so, instead of CE being part of a wider belief in how policy making should also be based on CE. This need for a shared vision was also experienced by the community-led initiative in region F. The remaining volunteers and the support worker started looking for what their next steps and new aims should be after the old governance board had left and the initiative was nearly disbanded (context). As the community-led initiative had nearly collapsed, it created a sense of urgency and commitment with the remaining volunteers to continue the initiative (mechanism). At the same time, they experienced it as difficult to rise above the failings and negative experiences (to “let go off the old ballast”; mechanism). This meant that they had not yet succeeded in developing a new vision and that they were still searching for a vision that could act as the connecting thread for the initiative (outcome):

It’s also about the colleagues...It matters how the process is handled and by who. There’s quite a big differences in that. We don’t have one clear view, vision or policy of “it’s in this way that we do CE or CE is always important in this phase of a project.” Of course CE is not a one-size-fits-all approach, but unknown makes unloved, I think. There’s so many people whereby CE is not part of the process.

Region A, policy maker, male

#### Theme 7: Misalignment Between Citizens’ and Professionals’ Perspectives and Motivations for CE

Fourth, and related to the lack of a shared vision, citizens and professionals had experienced a misalignment between citizens’ and professionals’ perspectives and motivations for CE and thus had different experiences throughout the process of CE. Citizens and professionals had experienced this lack of alignment in both organizationally led CE approaches and community-led initiatives. The citizens stated that they felt that professionals were too outcomes focused. For example, the community-led initiative in region F was in transition and was searching for which aims and activities should be continued and taken up (context). Engaged citizens and professionals had differing goals and ambitions (context). Professionals were more outcome focused, which citizens felt like made the initiative aim too high (mechanism). Citizens meanwhile were engaged because of their intrinsic motivations and because they wanted to increase their social connection within the community (mechanism). Such differences in aims should be openly discussed (outcome). Professionals in region D speculated what was underlying this misalignment. With CE approaches, everyone (citizens, professionals, and volunteers) involved has their own language, interests, and scope (context). Citizens often think and operate “on a smaller,” “community-based” level (context). Professionals become irritated because, from their perspectives and aims, they feel change is not happening fast enough (mechanism). The professionals felt this showed that motivations between citizens and professionals were not aligned and that resource investments (especially time and space) should be created to discuss these differences and to address the motivations and interests of citizens more specifically (outcome). This is comparable with citizens’ experiences who had also underscored the importance of creating a transparent dialogue between citizens and professionals to align the motivations:

Differences in interests...You have to have a shared goal.

Region F, citizen, female

### Reference Panel Deliberations

Panel members recognized the findings and stated they had also found it easier to involve citizens with local approaches, which were more aligned with citizens’ lived experiences. Furthermore, both citizens and professionals within the panel also underscored their search for new collaborative forms of working between citizens and organizations and how to best involve citizens in the decision-making process. For example, they were searching for ways to enable some citizens to be involved in the long term (mostly in governance structures) and at the same time allow other citizens to be involved in the short term (without too much investment of their time and effort). The panel also discussed important enablers to work toward these new ways of collaborative working for CE. For example, both citizens and professionals within the panel highlighted that one of the most important enablers was having leadership who can create support and garner interest for CE. The professionals particularly highlighted that such leadership would help to change the culture within organizations, for example, ensuring citizens are not involved because this has been decreed top-down (eg, through the Participation Act 2015 and Living Environment Act 2021) but because there is a sincere belief and hope within the organization to ensure services and policies are better aligned to citizens’ and communities’ needs and experiences. They also underscored the significant importance of a clear vision and corresponding plan for CE, for example, who should be involved, when, where, and about which topics. Finally, both citizens and professionals within the panel stated the importance of long-term investments to properly embed CE within their organizations or their neighborhoods. Citizens especially underscored their need to have organizations (health and care organizations and local and regional governments) invest financially within their initiatives in the longer term, whereas professionals stated that they needed the time and space to be able to innovate CE—not merely through financial investments but by being given more time and space to involve citizens and to experiment with new CE approaches and activities.

## Discussion

### Principal Findings

Using the RE approach, this multiple case study investigated how CE approaches in 6 different Dutch regions have changed over the past 4 years. It also investigated citizens’ and professionals’ underlying experiences impacting these changes. The results have shown that CE approaches are changing from having a region-wide focus to a more community-based focus, to building relationships with engaged citizens, and to focusing more on practical health promotion activities (rather than “abstract” topics such as the redesign of regional hospitals). The results of this study also suggest that CE (including the underlying understanding of how to develop and implement CE successfully) still has not been embedded within organizational cultures. This has arguably meant that the remaining CE approaches seem to be operating on a smaller scale (instead of using a mix of smaller scale and more regional approaches; Table 2) and that professionals and citizens required further investments in the engagement environment, the need for facilitative leadership, and the need for a shared vision on how to act upon CE based on aligned motivations.

Deductively analyzing the results showed if and how the guiding principles [13,14] (described in the *Methods* section) were being considered and applied within the 6 regions. The guiding principle that professionals were particularly concerned with was principle 1, which pertained to leadership. Professionals were aware that they needed to develop their facilitative leadership toward citizens and also required more supportive leadership from their organizations to better embed CE within projects and organizations. Broadly speaking, although many of the interviewed professionals observed and believed in the benefits of CE, they felt that their management largely did not. They felt this prevented the proper embedding of CE within organizational cultures and also hindered them from involving citizens as early as possible (principle 3) and often prevented them from sharing decision-making control with citizens (principle 4). Furthermore, citizens within this study often discussed the importance of open and transparent dialogue between citizens and professionals regarding their motivations and aims for CE approaches (principle 8). As Beresford [10] suggests, CE in health has been shaped by the political agendas of (national and local) politicians, policy makers, and professionals, and Willems [31] has shown that efficiency and effectiveness are important underlying CE aims for organizations, which has made it harder for organizations to deploy resources to improve and develop CE. Similar to previous studies, this study has shown that CE approaches (only) focused on organizational (regional and more abstract) aims largely failed to motivate citizens to become involved [11,13,14,32]. By openly discussing these aims and providing the space and leadership to communities to share their aims, CE approaches can hopefully better address citizens’ aims as well.

Relatedly, an important principle that participants had recognized and experienced as an important barrier but had not yet actively invested in was principle 9 regarding the development of a shared vision for CE. This may well be related to the experienced lack of supportive leadership and dialogue (between citizens and professionals), as described above. For example, citizens highlighted the importance of articulating achievable goals and highlighted the importance of transparently discussing any differences in aims. While professionals had experienced a lack of time to formulate clear and achievable goals for CE projects—perhaps because management felt like CE has been forced upon them by national policies like the Participation Act (2015) and the Living Environment Act (2021) as some professionals within this study had theorized.

Relatedly, one of the reasons for this lack of transparent dialogue between citizens and professionals regarding a CE vision could be the fact that both citizens and professionals described a lack of investment in the engagement environment as an important barrier. Such findings are in line with previous studies, such as the study by Holley [14], which has shown that many current engagement environments are built for efficiency, rather than, for example, building relationships with not-yet engaged or harder-to-reach groups. Such an engagement environment often results in a loss of influence for citizens, especially those who are socioeconomically disadvantaged [14]. This finding is further underscored by the fact that very few of this study’s participants had discussed experiences regarding the addressing of power imbalances (principle 5) or had discussed experiences regarding the need to develop safe and trusting environments for citizens to enable citizens’ involvement (principle 2). This study’s participants highlighted the importance of properly embedding CE, for example, by making CE a structural and routine part of projects and policy development; by providing citizens and professionals with the time and space to develop creative engagement approaches; by providing community-led initiatives with long-term financial support; and by helping professionals to develop CE skills and know-how, for example, by providing training and guidelines.

This lack of investment in the engagement environment, leadership, and shared vision (based on aligned citizens’ and professionals’ motivations) may well have led to organizations in the 6 regions choosing to shift from a regional approach to a community-based focus and shifting their focus from more complex regional topics to more tangible projects, instead of trying to bolster and improve the original approaches (through such investments) and at the same time also build relationships with communities and supporting more tangible projects. Arguably the different types of CE approaches (ie, regional, focused on complex issues such as the reconfiguration of health care services, community-based and focused on building relationships with communities, and focused on health promotion activities) should be applied alongside each other. Building relationships with citizens will also help to ensure CE approaches are better aligned with citizens’ lived experiences and motivations. Prior literature indicates that citizens exhibit diverse interests and preferences for involvement, ranging from engaging in practical activities and providing peer support to participating in policy-making processes to ensure that policies better reflect their lived experiences [32,33]. To enhance citizens’ more active participation in the development and delivery of health and care services, an investment is required to develop various types of approaches beyond the currently defined roles [16,17].

Despite the fact that this study indicates a systemic lack of investment in CE, this study also offers hopeful signs. First, Table 2 only shows the CE approaches that have been implemented and does not show potentially positive underlying (cultural) changes. For example, 1 citizen in region A described that they felt more collaboration was taking place between organizations and client councils. Furthermore, professionals within this study suggest that newer policy makers and professionals have been trained to believe in the value of CE and want to investigate new and more collaborative ways of working with communities and citizens. Not only has this newer generation been trained to believe in citizens’ and communities’ rights to be involved but also their CE experiences (with more local approaches) have shown them the benefits of involving citizens, for example, ensuring that policies are more aligned with citizens’ own experiences and needs [15]. Furthermore, this study’s findings also indicate ways to improve the engagement environment and to further develop CE. CE should be supported by a flexible system rather than bureaucratic systems and processes, which should be underpinned by a variety of creative CE approaches, sufficient resources (ie, know-how, time, and finances), and an organizational culture that maintains CE as “business as usual” for all projects. These findings suggest that a new guiding principle should be formulated regarding the different ways in which a supportive engagement environment can be implemented. More research is required to properly formulate this new guiding principle, though the results of this study show that such a principle should underline 3 different but interrelated aspects of CE. A supportive engagement environment requires (1) structural investment, including staff with CE know-how and skills, finances, and time and space to develop creative CE approaches; (2) facilitative leadership within and for communities and organizations; and (3) a clear and shared CE vision (based on alignment of citizens’ and professionals’ motivations). There is a circularity to the 3 aspects that makes it harder for organizations to know where to start when (further) developing their CE approaches. For example, leadership and an investment of resources may be required to create a shared vision for CE. However, a shared vision is also required to leverage sufficient resources and leadership at different levels within organizations and communities. Ultimately, this study suggests that without such investments, it will be challenging to fully integrate CE into organizational cultures and to transition CE from being perceived merely as a beneficial addition to health and care systems to being recognized as essential for enhancing transparency, accountability, equity, and person-centeredness within those systems.

### Limitations

One limitation is the relatively small number of participants, especially engaged citizens, for the primary data source (T4). Unfortunately, the first COVID-19 wave may have prevented more participants, working and volunteering in the health and care system, from taking part. This limitation was mitigated by the fact that this study tracked the CE approaches being implemented for 4 years and by the reference panel’s workshop discussions as this confirmed the validity and applicability of our interview findings in other contexts, thus further validating and enriching the interview findings. Another COVID-19–related limitation was the fact that interviews had to take place on the web or over the telephone; this prevented participants from fully reviewing their storyboards and areas for further development of CE.

### Future Studies

This case study indicates the importance of a supportive engagement environment created by structural investments, including staff with know-how and skills, finances, and space to develop creative CE approaches; facilitative leadership within and for communities and organizations; and a clear and shared overarching vision for CE based on the alignment of citizens’ and professionals’ motivations. However, future studies are required to further unpack these aspects of CE and to highlight how to practically apply these aspects for the improvement of CE. For example, future studies could focus on how to create a transparent dialogue between communities and organizations to align communities’ and organizations’ aims for CE. Future studies could also examine different (and more practical) ways in which the engagement environment can be improved and supported by organizational management and regional and national governments.

### Conclusions

This study investigated how CE approaches had changed over the past 4 years in 6 different regions in the Netherlands. It examined citizens’ and professionals’ experiences underlying these changes, including the barriers, enablers, and support needs. The study showed three overarching themes along which CE had been adapted: (1) moving away from regional CE approaches; (2) focusing on building relationships with already-engaged citizens and communities; and (3) focusing on practical, tangible health promotion activities (instead of more complex “abstract” programs). Furthermore, participants had experienced (1) a lack of a supportive engagement environment, (2) a lack of facilitative leadership, (3) a lack of a shared vision for CE, and (4) a misalignment in citizens’ and professionals’ aims. The study suggests that citizens and professionals perceive and experience CE differently and that they have different priorities for CE. To enable and support the further development of CE approaches, both citizens and professionals experienced the need for investments in the engagement environment (eg, through more structural organizational support, time, and space to innovate and improve CE approaches and to embed CE within organizational cultures), for more facilitative leadership, the need to develop a shared vision, and the alignment of citizens’ and professionals’ motivations. Such investments and changes to organizational cultures, structures, and processes would enable organizations to be more open and sensitive to the different ways in which different citizens want to be involved. Without such further investments and leadership, CE will remain seemingly smaller scale and piecemeal, instead of being seen as crucial to restoring accountability and person-centeredness to health and care systems.

## Appendix

Multimedia Appendix 1 Realist evaluation standard reporting form.

Multimedia Appendix 2 Reference panel participants’ description.

Multimedia Appendix 3 Summary of the context-mechanism-outcomes underpinning themes.

## Abbreviations

CE: community engagement
CMO: context-mechanism-outcome
RE: realist evaluation

## Data Availability

All data generated and analyzed during this study are included in the published article and supplementary information files. Templates used for data extraction and analysis are available from the corresponding author on reasonable request.

## References

[1] Carman KL, Dardess P, Maurer M, Sofaer S, Adams K, Bechtel C, Sweeney J (2013). Patient and family engagement: a framework for understanding the elements and developing interventions and policies. Health Aff (Millwood).

[2] O'Mara-Eves A, Brunton G, McDaid D, Oliver S, Kavanagh J, Jamal F, Matosevic T, Harden A, Thomas J (2013). Community engagement to reduce inequalities in health: a systematic review, meta-analysis and economic analysis. NIHR Journals Library.

[3] Barnes M, Knops A, Newman J, Sullivan H (2004). Recent research: The micro-politics of deliberation: case studies in public participation. Contemp Polit.

[4] Bruni RA, Laupacis A, Martin DK, University of Toronto Priority Setting in Health Care Research Group (2008). Public engagement in setting priorities in health care. CMAJ.

[5] Fung A (2015). Putting the public back into governance: the challenges of citizen participation and its future. Public Adm Rev.

[6] Glimmerveen L, Nies H, Ybema S (2019). Citizens as active participants in integrated care: challenging the field's dominant paradigms. Int J Integr Care.

[7] Tenbensel T (2010). Virtual special issue introduction: public participation in health policy in high income countries--a review of why, who, what, which, and where?. Soc Sci Med.

[8] Ocloo J, Matthews R (2016). From tokenism to empowerment: progressing patient and public involvement in healthcare improvement. BMJ Qual Saf.

[9] Rowe G, Frewer LJ (2016). A typology of public engagement mechanisms. Sci Technol Hum Val.

[10] Beresford P (2019). Public participation in health and social care: exploring the co-production of knowledge. Front Sociol.

[11] Cooke B, Kothari U (2001). Participation: The New Tyranny?.

[12] Boivin A, Lehoux P, Burgers J, Grol R (2014). What are the key ingredients for effective public involvement in health care improvement and policy decisions? A randomized trial process evaluation. Milbank Q.

[13] De Weger E, Van Vooren N, Luijkx KG, Baan CA, Drewes HW (2018). Achieving successful community engagement: a rapid realist review. BMC Health Serv Res.

[14] De Weger E, Van Vooren NJ, Drewes HW, Luijkx KG, Baan CA (2020). Searching for new community engagement approaches in the Netherlands: a realist qualitative study. BMC Public Health.

[15] Holley K (2016). The principles for equitable and inclusive civic engagement: a guide to transformative change. The Kirwan Institute for the Study of Race and Ethnicity.

[16] De Weger E, Drewes HW, Van Vooren NJ, Luijkx KG, Baan CA (2022). Engaging citizens in local health policymaking. A realist explorative case-study. PLoS One.

[17] de Weger E (2022). A Work in Progress: Successfully Engaging Communities for Health and Wellbeing. A Realist Evaluation.

[18] Wong G, Westhorp G, Manzano A, Greenhalgh J, Jagosh J, Greenhalgh T (2016). RAMESES II reporting standards for realist evaluations. BMC Med.

[19] Pawson R, Tilley N (1997). Realistic Evaluation.

[20] Lacouture A, Breton E, Guichard A, Ridde V (2015). The concept of mechanism from a realist approach: a scoping review to facilitate its operationalization in public health program evaluation. Implement Sci.

[21] Jagosh J, Macaulay AC, Pluye P, Salsberg J, Bush PL, Henderson J, Sirett E, Wong G, Cargo M, Herbert CP, Seifer SD, Green LW, Greenhalgh T (2012). Uncovering the benefits of participatory research: implications of a realist review for health research and practice. Milbank Q.

[22] Macfarlane F, Greenhalgh T, Humphrey C, Hughes J, Butler C, Pawson R (2011). A new workforce in the making? A case study of strategic human resource management in a whole-system change effort in healthcare. J Health Organ Manag.

[23] Jagosh J, Bush PL, Salsberg J, Macaulay AC, Greenhalgh T, Wong G, Cargo M, Green LW, Herbert CP, Pluye P (2015). A realist evaluation of community-based participatory research: partnership synergy, trust building and related ripple effects. BMC Public Health.

[24] Astbury B, Leeuw FL (2010). Unpacking black boxes: mechanisms and theory building in evaluation. Am J Eval.

[25] RAMESES project.

[26] Mays N, Pope C (1995). Rigour and qualitative research. BMJ.

[27] Warwick-Booth L, Cross R (2016). Using storyboards in participatory research. Nurse Res.

[28] Lupton D Doing fieldwork in a pandemic (crowd-sourced document). Google Docs.

[29] Colucci E (2007). "Focus groups can be fun": the use of activity-oriented questions in focus group discussions. Qual Health Res.

[30] Giacomini MK, Cook DJ (2000). Users' guides to the medical literature: XXIII. Qualitative research in health care A. Are the results of the study valid? Evidence-based medicine working group. JAMA.

[31] Willems W (2021). Countering the tragedy of the health care commons by exnovation: bringing unexpected problems and solutions into view. Sustainability.

[32] De Weger E, Baan C, Bos C, Luijkx K, Drewes H (2022). 'They need to ask me first'. Community engagement with low-income citizens. A realist qualitative case-study. Health Expect.

[33] Schmidthuber L, Piller F, Bogers M, Hilgers D (2019). Citizen participation in public administration: investigating open government for social innovation. R D Manag.

