# Supplementary material for: Tracking the Development of Community Engagement Over Time: Realist Qualitative Study
Source: J Particip Med. 2024 May 15;16:e47500. doi: 10.2196/47500 (PMC11137424; doi:10.2196/47500)
Supplement: Multimedia Appendix 1 [file jopm_v16i1e47500_app1.docx]

**Appendix III Realist Evaluation Standard Reporting Form**

| **Title** | | **Reported in document Y/N/Unclear** | **Page(s) in document** |
| --- | --- | --- | --- |
| 1 | In the title, identify the document as a realist evaluation | Yes | The realist evaluation is mentioned in the title |
| **Summary or abstract** | | | |
| 2 | The abstract should include brief details on: the policy, programme, or initiative under evaluation; programme setting; purpose of the evaluation; evaluation question(s) and/or objective(s); evaluation strategy; data collection; documentation and analysis methods; key findings; and conclusions | Yes | Page 1. The abstract includes a description of the policies and programmes under evaluation, purpose of the evaluation, the research question, data collection and analysis methods, the key findings and conclusions. |
| **Introduction** | | | |
| 3 | Rationale for evaluation: explain the purpose of the evaluation and the implications for its focus and design | Yes | The introduction introduces the RE approach. But in the methods section the rationale for choosing the RE method are explained as well as the implications for the study focus (and design):  *The study was informed by the realist evaluation (RE) approach. The RE approach seeks to explain the causal relationship between contexts, mechanisms and outcomes in particular programmes of interest (Pawson & Tilley 1997). In this way, the study sought to understand the causation behind the changes in CE approaches and to understand which (enabling and constraining) mechanisms were triggered within the (changing) contexts of the six regions and how these influenced citizens’ and professionals’ experiences of developing CE. (See Table I for CE-oriented definitions of realist concepts)*, p.4. |
| 4 | Programme theory: describe the initial programme theory (or theories) that underpin the programme, policy, or initiative |  | The programme theories that underpin the study are described in the methods section, under the data analysis subsection. This section also provides an overview of how these guiding principles were developed:  *These guiding principles are: (a) ensure staff provide supportive and facilitative leadership to citizens; (b) foster a safe and trusting environment enabling citizens to provide input; (c) ensure citizens’ early involvement; (d) share decision-making and governance control with citizens; (e) acknowledge and address citizens’ experiences of power imbalances between citizens and professionals; (f) invest in citizens who feel they lack the skills and confidence to engage; (g) create quick and tangible wins; (h) take into account both citizens’ and organisations’ motivations; (i) develop a shared vision with clear roles for professionals and citizens, ensuring communities’ diversity is reflected within the vision.* Page: 11. |
| 5 | Evaluation questions, objectives, and focus: state the evaluation question(s) and specify the objectives for the evaluation. Describe whether and how the programme theory was used to define the scope and focus of the evaluation |  | - The research objectives, questions and focus are described in the introduction: pages 3-4 - In the methods section: page 4. - The description and application of the programme theories is described in the introduction on pages: 3-4 - And in the methods section on page: 11. |
| 6 | Ethical approval: state whether the realist evaluation required and has gained ethical approval from the relevant authorities, providing details as appropriate. If ethical approval was deemed unnecessary, explain why. |  | Ethical approval is described on page 12 and on page 29.  Ethical approval was obtained through Tilburg University Ethical Board |
| **Methods** | | | |
| 7 | Rationale for using realist evaluation: explain why a realist evaluation approach was chosen and (if relevant) adapted |  | The rationale for applying the realist evaluation approach is described in the methods section on page 4. *See above* |
| 8 | Environment surrounding the evaluation: describe the environment in which the evaluation took place |  | The wider context in which the evaluation took place is described in the introduction section on pages: 3-4  And in the methods section with Table II on pages 7-10 |
| 9 | Describe the programme policy, initiative or product evaluated: provide relevant details on the programme, policy or initiative evaluated |  | The study is focusses on the development of CE in six different regions in the Netherlands and such does not focus on only one initiative or policy. This is described throughout the paper, including in the introduction, methods, and results. |
| 10 | Describe and justify the evaluation design: A description and justification of the evaluation design (i.e. the account of what was planned, done and why) should be included, at least in summary form or appendix, in the document which presents the main findings. If this is not done, the omission should be justified and a reference or link to the evaluation design should be given. It may also be useful to publish or make freely available (e.g. online on a website) any original evaluation design document or protocol, where they exist |  | The evaluation design is especially described in the methods section with an account of:   - Realist evaluation approach - Recruitment approach - Data collection - Data analysis - And the inclusion of the reference panel to help ensure the rigour, validity and trustworthiness of the data. - Additionally, the interview topic guide and coding tree are supplied in appendices   These descriptions highlight how the realist evaluation methods was taking into account in every step of the study (especially in the data collection, analysis and the reference panel). |
| 11 | Data collection methods: Describe and justify the data collection methods—which ones were used, why and how they find into developing, supporting, refuting, or refining the programme theory. Provide details on the steps taken to enhance the trustworthiness of the data collection and documentation |  | In the methods section the data collection methods are described on page 10.  The methods section describes how the interviewees were asked to complete storyboards to enable their reflexivity on the development of their CE approaches.  The steps taken to ensure the trustworthiness, validity and rigour of the data is also described in the methods section. This section highlights that:   - data had been collected to the point of data saturation (page: 6) - the coding had been discussed and refined with the entire research team (Page: 11-12) - a reference panel workshop was conducted to discuss, check and refine the preliminary findings (pages: 6-7 and pages 23-24). - data triangulation between the data sources was conducted (page: 11) |
| 12 | Recruitment process: describe how respondents to the evaluation were recruited or engaged and how the sample contributed to the development, support, refutation or refinement of programme theory |  | The recruitment process is described on page 6. |
| 13 | Data analysis: describe in detail how data were analysed. This section should include information on the constructs that were identified, the process of analysis, how the programme theory was further developed, supported, refuted and refined and (where relevant) how analysis changed as the evaluation unfolded |  | The data analysis is described on page 11. How the analysis process refined the programme theories is described in the discussion section (page 25). |
| **Results** | | | |
| 14 | Details of participants: report (if applicable) who took part in the evaluation, the details of the data they provided, and how the data was used to develop, support, refute or refine programme theory |  | The interview participants and the reference panel participants are described in Table II on pages (7-10). |
| 15 | Main findings: present the key findings, linking them to contexts, mechanisms, and outcome configurations. Show how they were used to further develop, test or refine the programme theory |  | The key themes and the CMOs are described throughout the results section. Additionally a summary of CMOs is presented in an Appendix II. |
| **Discussion** | | | |
| 16 | Summary of findings: summarise the main findings with attention to the evaluation questions, purpose of the evaluation, programme theory, and intended audience |  | At the beginning of the discussion the findings are summarized. Consequently the purpose and the adapted programme theories are discussed (page: 25). |
| 17 | Strengths, limitations, and future directions: Discuss both the strengths of the evaluation and its limitations. These should include (but need not be limited to): (1) consideration of all the steps in the evaluation processes; (2) comment on the adequacy, trustworthiness and value of the explanatory insights which emerged. In many evaluations, there will be an expectation to provide guidance on future directions for the programme, policy or initiative, its implementation and/or design. The particular implications arising from the realist nature of the findings should be reflected in these discussions |  | Throughout the discussion the programme theories are discussed, including the realist nature of these programme theories (pages 25-26).  The limitations are discussed on page 28. |
| 18 | Comparison with existing literature: where appropriate, compare and contrast the findings with the existing literature on similar programmes, policies or initiatives |  | Throughout the discussion, the previous literature is used to compare and contrast the findings. Some of this previous literature is based on previous realist findings. |
| 19 | Conclusion and recommendations: List the main conclusions that are justified by the analyses of the data. If appropriate offer recommendations consistent with a realist approach |  | The conclusion highlights the main themes and provides recommendations to further develop CE in the Netherlands successfully and meaningfully. |
| 20 | Funding and conflicts of interest: state the funding source (if any) for the evaluation, the role played by the funder (if any) and any conflicts of interests of the evaluators |  | (Lack of) funding and conflicts of interest is mentioned on page 30. |
