# Supplementary material for: Tracking the Development of Community Engagement Over Time: Realist Qualitative Study
Source: J Particip Med. 2024 May 15;16:e47500. doi: 10.2196/47500 (PMC11137424; doi:10.2196/47500)
Supplement: Multimedia Appendix 2 [file jopm_v16i1e47500_app2.docx]

**Appendix I: Reference panel participant description**

| Nr. | Type of organization | Type of function |
| --- | --- | --- |
| 1. | Community-led initiative | Volunteer, community-led initiative board member |
| 2. | Community-led initiative | Volunteer, community-led initiative board member |
| 3. | Community-led initiative | Volunteer village key worker, community-led initiative board member |
| 4. | Patient & Public Involvement organization | Representative role, outreach role |
| 5. | Patient & Public Involvement organization | Representative role, project management role |
| 6. | Patient & Public Involvement organization | Representative role, educational role for both citizens & organisations |
| 7. | Patient & Public Involvement organization | Representative role, policymaker |
| 8. | Municipality | Policymaker |
| 9. | Municipality | Policymaker |
| 10. | Municipality | Policymaker |
| 11. | Municipality | Policymaker |
| 12. | Health & care organization | Public health professional |
| 13. | Knowledge institutes | Researcher |
| 14. | Knowledge institutes | Researcher |
| 15. | Knowledge institutes | Researcher |
| 16. | Knowledge institutes | Researcher |
| 17. | Knowledge institutes | Commissioner of research |
