# Supplementary material for: Tracking the Development of Community Engagement Over Time: Realist Qualitative Study
Source: J Particip Med. 2024 May 15;16:e47500. doi: 10.2196/47500 (PMC11137424; doi:10.2196/47500)
Supplement: Multimedia Appendix 3 [file jopm_v16i1e47500_app3.docx]

**Appendix II: Summary of CMOs underpinning themes**

| **RQ1: Changes in CE approaches** | | | | | |
| --- | --- | --- | --- | --- | --- |
| **Theme: From regional to community focussed** | | | | | |
|  | **Context** | | **Mechanism** | | **Outcome** |
| 1. | - Municipality is made up of 13 smaller communities - Municipality had originally started their CE approach focussing on municipality-wide events. Moreover, these events were organised when and where it was convenient to the municipality | | - However, citizens did not feel connected to the wider municipality; they only felt a connection to their own community. - This disconnect meant that citizens were not motivated to take part in municipality-wide events or projects | | That is why the municipality eventually decided to move away from municipality-wide approaches and started focussing on the communities instead, e.g. by building relationships with the village councils, by attending meetings/events that the communities were organising themselves |
| 2. | Municipality developed a community-focussed (instead of regional approach) as they had experienced the regional approach was unsuccessful | | Despite the fact that the community-focussed approach is more intensive and requires more time investments, citizens feel more connected to their own community and feel more motivated to participate regarding one clear subject | | This new community-focussed approach has led to more connection and involvement from citizens |
| 3. | This new community-focussed approach was first tested through a youth worker who had held events and interviews with young people in their own communities; as this had proven successful the community is now building on from that CE approach | | The cornerstone of this new approach is to base CE approaches on the issues where citizens feel a sense of urgency | | That is why the new CE approach is focussed on the activities and happenings within the communities |
| 4. | - Positive Health Network tried to develop a CE approach based on the Positive Health concept on a regional level. - Now they are starting to develop a community-based approach and a village council has been engaged | | There has been a lot of attention (from the department of health, health care insurance companies) which motivates citizens and professionals to get involved | | The Positive Health Network is wanting to build on the successful experience of this first community-focussed approach (giving citizens a more active role as well) |
| 5. | A charismatic GP connected to the Positive Health Network was successful in engaging citizens in implementing Positive Health within one village in the region | | Citizens within this village were motivated to participate due to the sense of urgency created by the greying population within the village and because many (younger) people are leaving the village | | This has led the Positive Health Network to consider changing their CE approach from regionally-focussed to more community/village-focussed |
| 6. | Positive Health Network has experienced that regional approaches based on more abstract health care system issues are not successful for CE | | Based on this experience, they know think it is important to leverage key figures from a community to motivate other citizens to get engaged. Such a key figure will also be more invested in the community and be less inclined to give up on CE | | - Such a key figure has a clearer view on how the community operates and see the value of engaging other citizens - Positive Health Network is therefore trying to develop a more community-based CE approach |
| 7. | The first, regional, CE approach based on the Policyholder Cooperation had been experienced as unsuccessful | | - The Cooperation concerned itself with complicated, regional issues (like the regional economy and the viability of the hospital). The board felt that these issues were too complex to motivate ‘average citizens’ - That is why some professionals/organisations in the board want to move from the regional approach to more community-focussed approach | | - The regional board is therefore looking to involve and facilitate citizens in practical health promotion activities aimed at improving the health and social cohesion of communities - Board is now searching for ways to develop CE in a way that it is more connected to communities |
| 8. | Original regional CE approach (the Policyholder Cooperation) was experienced as unsuccessful. Because they ostensibly wanted to continue developing CE in the region, the regional health and care board decided look for other ways to connect with citizens to anchor the culture of CE | | Local councillors and project managers on the regional health and care board are still hopeful that CE can be anchored within cultures, but feel this anchoring of CE is best achieved through a community-based approach | | The board is still looking how they can facilitative communities to take up CE themselves. |
| 9. | Original regional CE approach (the Policyholder Cooperation) was experienced as unsuccessful. Because they ostensibly wanted to continue developing CE in the region, the regional health and care board decided to involve citizens in the organisation of health promotion activities | | The Board hopes to ensure citizens in the future know and can come to organisations with their ideas for help and support and therefore wish to build relationships with communities | | - The Board wants to move to a more community-based approach whereby they facilitate motivated citizens to develop and implement their own projects - But the Board has no clear picture of how they can help build and facilitate such community power/action |
| 10. | - Original regional CE approach (the Policyholder Cooperation) was experienced as unsuccessful, they were looking for new CE approaches which were centred on facilitating communities’ own ideas and projects. - Health care board experienced that CE projects are more easily taken up and developed in the villages rather than the cities in the region. | | The regional health and care board expect that it is easier to start CE approaches in smaller villages, because of its smaller scale as there is more social cohesion and residents know each other (better) | | - The Board is now looking how community power/action can be facilitated - They are hoping to leverage support workers and youth workers as a linking pin and to build relationships with communities |
| **Theme: building relationships** | | | | | |
| 11. | Originally, (local) governments knew what was best for communities. However, policymakers have had positive experiences of involving citizens in developing and renewing communities’ social spaces | | Through these positive experiences he’s started thinking differently about CE and is more motivated to involve citizens during the design phase and see CE more as an iterative process | | While policymakers would originally ‘just’ organise meetings with citizens to inform them of plans, policymakers and the municipality have seen the importance of building relationships with communities and citizens |
| 12. | First time policymakers started a CE project about the healthy living environment by opening up dialogue with residents | | It was difficult for municipality to share a certain control (‘to let things go’) due to the fear that citizens would highlight issues or share ideas that the municipality would not want to tackle | | Due to this ongoing dialogue between the municipality and citizens, both parties understood each others perspectives better |
| 13. | - The original/older policymakers see the municipal as in the lead and do not see the value of CE (think the opinion of citizens is irrelevant and that CE costs too much time and resources) - New generation of policymakers have a different view of CE and see the value of involving citizens | | Successful CE projects do not seem to motivate the older generation of policymakers to adjust their view on CE, as they struggle to share control with citizens | | - That is why the required cultural change takes so much time - And why CE projects are not always successful: if relationships are not built with citizens, if citizens are not involved early on, citizens will not feel heard. |
| 14. | Primary Care Group had unsuccessful experiences regarding the developing and implementing of CE as they did not have a clear vision for CE | | The organization should wish to first build relationships with citizens to create trust and goodwill | | Only then can (shared) a vision be developed (including goals, citizens’ roles and level of influence, expectations). |
| 15. | - After the regional Health and Care Board experienced the original regional CE approach (the Policyholder Cooperation) as unsuccessful, meant that they were now looking for new ways to support citizens to develop their own CE approaches - Now looking for different ways and routes to raise awareness with citizens that they can solve issues within their own communities themselves (e.g. through local media) | | The Board realizes that the known routes do not work for low-income neighbourhoods and are therefore looking for ways to build relationships with such neighbourhoods | | They have started leveraging support workers and youth workers to act as a link between the Board and the low-income neighbourhoods |
| 16. | - After the regional Health and Care Board experienced the original regional CE approach (the Policyholder Cooperation) as unsuccessful, they had started to develop a more facilitative approach to CE at least with the communities where there is already community action taking place | | The Board wanted to build relationships with communities, thus hoping to create a connection with communities and to stimulate a sense of co-ownership of CE for communities | | The Board in this way hoped to better align CE approaches to what motivates citizens themselves. |
| **Theme: practical/tangible health promotion activities (instead of more complex ‘abstract’ programmes)** | | | | | |
| 17. | It is still unclear to the municipality when and how citizen participation should be implemented as part of projects; i.e.: should citizens only be involved when there is a concrete/clear project or to ensure citizens and village councils feel heard | | Policymakers experience this as a difficult balance as they feel it is important to ensure citizens feel heard, but not if it disrupts concrete projects. | | Policymakers are considering taking time (away from specific projects) to build relationships with citizens/village councils to ensure they feel heard and at the same time to ensure it does not disrupt projects |
| 18. | Community-led initiative had organized the ‘living library’ (with the aim of increasing social cohesion and decreasing social division) | | By ensuring different types of people can share their story and experiences, it helps residents to appreciate the perspectives of people completely different from themselves | | The living library has proven very successful which is why the community-led initiative wants to keep organizing such practical events after the COVID-19 pandemic |
| 19. | Community-led initiative organized a lot of practical projects and events | | These practical projects and events were successful because there was a dedicated lead who knew how to motivate others | | This made these activities popular and made it clear that it was important to have one lead for each of the activities and projects |
| 20. | Community-led initiative had developed a health and wellbeing app together with the community college | | A lot of time and energy was put into the development of this app which turned out unsuccessful which was demotivating to the volunteers | | The app project was not aligned with the (older) volunteers’ intrinsic motivation. Volunteers are more invested in practical and tangible projects which visibly improve social cohesion within the community |
| 21. | - New CE approach within the municipality is to engage citizens on the topics they are most interested in themselves (more concrete/tangible issues) - Municipality has experienced it as difficult to motivate and involve citizens about complex/abstract policy issues | | The municipality has involved citizens on the basis of their own interests/passions and on the basis of citizens’ expertise. But municipality fears a conflict of interest if citizens are too often engaged on the basis of their expertise. Municipality fears a tension between the legal requirement for participation and the role of the municipality, the municipal resident council. | | This means that within this new approach, the municipality prefers to engage citizens for concrete/tangible projects based on their interests (“easier to keep everyone to their own specific role”) |
| 22. | Policymakers experienced the previous municipality’s approach to CE as too focused on abstract policy issues as the meetings on the abstract topics were not well attended | | Due to this negative experience, the municipality started searching for new CE approaches which would provide citizens with the sense that they are engaged/have more decision-making control | | That is why they are changing their CE approach by focusing more on the community, by focusing on more practical/tangible projects, and communicating better with citizens |
| 23. | Community-led initiative is in transition and the support worker and volunteers are looking for a new vision and new activities to organise | | - Citizens are motivated to organise practical activities as these deliver tangible outcomes. - While the support worker wants them to collaborate with the district nursing team for example | | - The practical activities and projects are successful - Mismatch between support worker & the volunteers - Moreover, district nursing team and the community-led initiative are not aligned and the district nursing team does not understand the added-value of the initiative. |
| 24. | - Apart from the Primary Care Group client council, the organization also started a Positive Health network, whereby both professionals and volunteers were involved - Network had organized activities aimed at promoting positive health (e.g. like workshops at libraries) | | Volunteers were interested in positive health and were motivated by the more practical/tangible workshops because with abstract projects they cannot see what happens with their input | | This is why the Council was disbanded and the practical activities kept going |
| 25. | With complex health care system issues (e.g. integrated care), it is difficult to engage citizens for Primary Care Group (who are focussed on improving primary care and implementing integrated care) | | With practical CE projects based on tangible issues, it is easier to involve citizens. Abstract health care system issues do not motivate citizens to get involved | | This makes it harder for Primary Care Group to develop CE approaches or to involve citizens |
| 26. | - Citizen involvement in the Positive Health network is centred on practical/tangible activities as more abstract health system issues are seen as too difficult - For the more abstract health system issues, ‘professional-citizens’ (those who work or have worked in the healthcare system) are engaged | | - Primary Care Group expects that these more abstract issues are not motivating/interesting to ‘normal citizens’ and that there is a difference in language and perspective between ‘professional-citizens’ and ‘normal citizens’. - This also leads to Primary Care Group to question how representative engaged citizens are (devaluing their involvement in the process) | | This means that Primary Care Group is still searching for ways to motivate/involve ‘normal-citizens’ and |
| 27. | Five years ago, the community-led initiative was developed | | The initiative managed to motivate a large group of citizens to participate in promoting positive health within the community | | That motivation was translated to practical activities and projects, like walking groups, healthcare markets, social groups to exchange their experiences. |
| 28. | Five years ago the community-led initiative was launched based on the concept of Positive Health and with the idea that there should not be a ‘one-size-fits-all’ approach to citizens’ involvement | | Large group of citizens were motivated to join initiative as they felt a connection with Positive Health and because the initiative enabled citizens to join however they preferred/matching their interests | | This motivation and preferred engagement options translated into a lot of practical, health promotion activities like walking groups and healthcare markets |
| 29. | - Community-led initiative started by charismatic healthcare professional - After the initiative managed to engage a lot of like-minded people, but struggled to raise awareness of Positive Health in the broader community | | The fact that the initiative included a lot of professionals and ‘like-minded people’ changed the dynamic of the initiative and made it feel more like another ‘business/organisation’ as the focus shifted away from more concrete/tangible projects and activities | | This change in dynamic demotivated a lot of engaged citizens and caused issues for the initiative |
| 30. | - Community-led initiative is in transition and new governance board is considering how to create new plans and communicate with external stakeholders - Because of the COVID-19 pandemic a lot of practical activities were no longer possible. | | This led to many engaged citizens to no longer feel a connection to the initiative as they were motivated by the social connections these activities created for them | | - The governance board is therefore looking how to renew these activities (in a COVID-19 safe way) and how to communicate these better to external stakeholders/citizens - They are also looking for the best approach to engage the citizens who miss the tangible projects/social activities (during a pandemic) |
| **RQ2: Experiences underlying changes in CE approaches** | | | | | |
| **Theme: (lack) of engagement environment** | | | | | |
|  | | **Context** | | **Mechanism** | **Outcome** |
| 1. | | - Organisational culture used to be that the municipality decided everything, but due to new laws like the Participation Act (2015) and the Living Environment Act (2021), they are forced to review the role citizens have - Newer generation of policymakers have been trained to see the value of CE | | Increasingly policymakers are seeing and believing the value of CE, but this belief is not supported by the wider municipality/their management | This slows down the cultural change required for successful CE |
| 2. | | Community-led initiative was able to organise activities successfully, despite the fact that there was no long-term investment for these activities | | The volunteers experience the organisation of such activities as draining: it costs them a lot of time and energy | This makes it difficult for the community-led initiative to ensure they can keep organizing the activities in the long-term without the required financial investments |
| 3. | | Community-led initiative is in transition and is searching for a new vision and new aims | | Volunteer wants to involve a broader range of citizens and wants to include their wishes as well | But for more residents to be able to join the initiative, requires an investment in the skills and knowledge of residents |
| 4. | | Community-led initiative is in transition which is why a lot of volunteers quit | | The reduced number of volunteers puts a lot of pressure on the remaining volunteers as they feel it costs them extra time and effort to keep the initiative going | That is why the remaining volunteers require help and support from professionals and the municipality |
| 5. | | Municipality changed its approach from municipality-wide to more community-focussed, facilitative | | The municipality has increasing younger policymakers working there who are less bureaucratic and feel it is natural to involve citizens as equal partners | This helps to speed up the required cultural change and to change the engagement environment |
| 6. | | Due to the positive experiences of a new more community-focused approach, the municipality has provided policymakers more resources (time and resources) | | Policymakers have started seeing CE as a cornerstone to successful policymaking and want to develop policy in collaboration with citizens. They have started believing in the value of collaboration between citizens and policymakers | They have started trusting in the lived-experience knowledge of citizens which has led to a better end result for projects |
| 7. | | - Community-led initiative is in transition and volunteers and support worker are looking for a new vision and new activities - They tried to set up a collaboration with care organisations and the district nursing team, but this failed | | - Health and care organisations are focussed on reducing socio-economic inequalities and therefore did not understand the value of the community-led initiative or the white, middle-class volunteers - Because the health and care organisations were not supportive of the initiative nor invested in the initiative (e.g. because they put a lot of terms around the collaboration like raking in subsidies), citizens became demotivated | This lack of engagement environment/collaboration between the initiative and health and care organisations highlighted the importance of aligning motivations and investing and appreciating community-led initiatives. |
| 8. | | Initiative nearly collapsed after the old governance board quit | | Only a few die-hard volunteers remained because of their passion and sense of urgency for the initiative | However, the initiative needs to invest in attracting new volunteers with a different mix of skills and interests |
| 9. | | Primary Care Group had applied for subsidy to involve organisations from cultural/creative sector to develop new and innovative ways to involve citizens (with the Positive Health Network) | | Because when only health and care organisations think about CE, they end up involving citizens in the traditional (more limited) way | The subsidy was rejected which means that this search remains (though they still have the ambition to develop creative engagement approaches) |
| 10. | | Primary Care Group has experienced it as difficult to develop a vision for CE | | Because citizens and professionals have different priorities and motivations, professionals still do not believe in the value of citizen involvement | At the moment professionals experience that they have too little time and space to develop a CE vision for the organisation |
| 11. | | CE has never been a priority for Primary Care Group and they have never had a vision for CE | | In order to develop a CE vision, they require space and time, and a colleague who prioritises CE above all else | Only then can a cultural change be instigated within the organization that is supportive of CE |
| 12. | | After the unsuccessful client council, Primary Care Group is now looking for a new CE approach, however they have not yet asked citizens themselves how they would wish to be involved | | Primary Care Group is not motivated to involve citizens yet, as they feel they do not have the required capacity for this | They lack the leader and resources to create a clear picture of how they should develop CE, without such an investment they do not want to engage citizens yet |
| 13. | | Community-led initiative was started five years ago with the aim of raising awareness of Positive Health in the Community and to ensure a wide range of professionals and citizens were involved | | While a large group of citizens was motivated to join the more practical/health promotion activities, the interviewee doubts very much whether the municipality and the wider community believes in the strength and added value of the community-led initiative | Interviewee feels that this has prevented the successful collaboration between the initiative and health and care organisations and feels an investment in this collaboration is needed to move forward |
| 14. | | - Community-led initiative was launched purely on the basis of citizens’ motivation and good will. Financial resources were not provided - Initial governance board had applied for financial investments and recognition from the municipality and were lobbying local councillors | | The municipality refused to provide any resources without a clear business plan from the initiative. This demotivated the initial board members significantly | That is why they quit the initiative, which in turn caused the initiative to almost be disbanded |
| 15. | | The community-led initiative’s new governance board delved into the financial situation of the initiative | | The initiative had created a solid financial buffer which provided the new governance board with a sense of space and possibility | This enables the initiative to develop some new ideas and projects |
| 16. | | Top-down structures for CE are more comfortable for administrators and organisations | | Administrators like CE being more formal as it keeps projects within their sphere of influence and more predictable for them. But for citizens such formal structures can be demotivating | The regional health and care board will have to invest in learning the lessons from the previous top-down CE approaches to develop these new approaches |
| 17. | | Through the unsuccessful experience of the Policyholder Cooperation, the Board learned the importance of building relationships with communities directly | | - But administrators find it difficult to make citizens’ priorities and building relationships a priority as they have their own projects and SMART goals. - CE often does not have SMART/measurable goals which is why administrators need to have guts and show actual leadership to build relationships, align goals and share control with citizens | However, within the region, administrators still need to invest in this cultural change to enable CE in the region |
| **Theme: search for facilitative leadership from organisations** | | | | | |
| 18. | | - With the municipality’s ‘traditional’ CE approach, citizens were involved when the municipality had a finished plan to present (and citizens were involved once the plan had been developed because they were expected to showcase CE) - The first time the municipality had tested this new approach of involving citizens from the start, was when they posed the new subsidy rules to citizens. - At first citizens were not sure what their role was as they were not sure what their role would be as being involved from the start meant they were required to do more than just provide feedback | | However, citizens became motivated when they experienced that being involved from the start meant they shared more decision-making control with the municipality. | Because of these successful experiences, municipality started looking at how they could provide citizens more facilitative leadership from the start (instead of already developing plans and projects before involving citizens) |
| 19. | | New village council had started enthusiastically on topics to do with youth care and young people with a good governance board | | When the village council organises something the other citizens are enthusiastic which creates commitment | - This means citizens organise a lot of activities - Municipality has learned that it is a more successful CE approach to facilitate such citizen involvement and to support the activities that citizens themselves organise than to organise formal/region-wide CE |
| 20. | | Municipality’s approach to CE is changing, previously they only put in time and effort to motivate citizens to come to meetings | | But once the municipality had received their feedback, the municipality became less motivated to keep involving citizens and to keep communicating with them | The municipality is trying to be more facilitative to citizens during the entire duration of projects, not just while they require their input (because the expectation is that ‘they’ll need citizens for the next project’ too) |
| 21. | | - The negative experiences of involving citizens when the municipality had already developed the plan, meant they started searching for a new approach to CE - The new approach is based on sharing the problem/issues municipality is trying to address with communities with the aim of improving the collaboration between engaged citizens and organisations | | The sharing of the problems/issues creates commitment with engaged citizens and organisations and motivates both the think about solutions/projects. | Through this new, more facilitative approach, everyone (municipality, engaged citizens, organisations) have gained more understanding of each other |
| 22. | | - Support worker started supporting the community-led initiative just as it was about to collapse. - A new vision and governance board was needed to take the next steps | | The support worker has always taken a facilitative approach hoping that the remaining volunteers would create their own vision, but the support worker noticed that they were looking to them to create the new vision and highlight the next steps | That slowly changed until finally one of the volunteers took on the leadership roles themselves |
| 23. | | - After an enthusiastic start, the initiative started looking for the most fitting governance structures - They chose a governance structure that separated the different projects | | This meant that the citizens did not feel connected to the whole initiative | They missed clear leadership to connect all engaged citizens which led to some citizens leaving the initiative |
| 24. | | - Community-led initiative was launched five years ago with a local healthcare professional in the lead but without a clear governance or leadership structure - Initially four board members were selected but most were health care professionals within the community as well | | Citizens felt these members were unapproachable and the healthcare professional who had launched the initiative was not motivated to take up the leadership role long-term | This lack of clear leadership made it difficult for the engaged citizens to know what the decision-making process was or who to turn to with their project ideas |
| 25. | | The community-led initiative had been without clear leadership or governance structure for a long time before the new board was initiated | | The engaged citizens really missed this lack of leadership and structure (felt it was a big barrier to their engagement) | That is why the new governance board wants to:   - Transparently discuss the required structure and what roles everyone should have - Receive (financial) resources and support from organisations - Discuss transparently what everyone’s goals and ambitions are for the initiative |
| **Search and need for the implementation of a clear and shared vision underscoring the importance of CE** | | | | | |
| 26. | | Successful experience of designing village square together with village council | | The positive experience motivated policymaker to ensure CE becomes part of a broader vision (rather than being conducted on ad hoc basis) | - Policymaker was able to build relationships with the community - Policymaker developed new CE skills and wants to apply those to new projects going forward |
| 27. | | - Policymakers’ old habits die hard (not sharing control with citizens) - There is not one clear/shared vision for CE within the municipality | | This has prevented policymakers from experiencing CE as part of their ‘day-to-day’ business: “unknown is unloved” | That is why the required cultural change to make CE successful takes a long time |
| 28. | | There is no clear/shared vision for CE within the municipality | | The municipality is still unconvinced that CE is of added value to them. The municipality is still searching for when citizen participation is useful to them | That is why CE is still often unsuccessful as citizens are often only involved so the municipality can showcase that they involved citizens (not because it is part of a wider vision) |
| 29. | | - Public health organisation client support worker started supporting the community-led initiative three years ago at a point when the initiative was about to collapse - Support worker was tasked with ensure vulnerable/harder-to-reach citizens would join the initiative, while the initiative volunteers were all white, middle-class people - This misalignment in motivations made that the support worker and the volunteers had spent a lot of time searching for a new vision. | | By spending so much time with the volunteers, she learned to appreciate the input and effort of the white, middle-class volunteers | That is why the support worker adjusted her role to supporting and facilitating the engaged citizens (rather than taking the initiative over from them) |
| 30. | | - Because the community-led initiative had nearly finished, the remaining volunteers & support worker started looking for what their next steps should be - They took a long time searching for that new vision | | Because the community-led initiative had nearly collapsed, this created a sense of urgency and commitment with the remaining volunteers to continue the initiative. At the same time, they experienced it as difficult to rise above the failings and negative experiences, to ‘let go off the old ballast’ | This meant that they had not yet succeeded in developing a new vision and they are still searching for connecting thread for the initiative. |
| 31. | | Primary Care Group started their client council and formed it very traditionally with lots of policies and meetings for them to review and take part in. Furthermore, there were very few members taking part in the council | | Organisation had never developed a clear vision for the council/CE and kept searching for ways to involve the council and for ways they could use their input | Because of this lack of vision (and without clear roles, ambitions) Primary Care Group disbanded the council. |
| 32. | | Primary Care Group does not have a legal requirement to involve citizens or to develop client councils. Because their mission is to support general practices, it is also not clear who their primary target group for CE would be | | Without this legal pressure, Primary Care Group never took the time to develop their own vision for CE | This also meant that other (organizational) issues always took priority over the engagement of citizens. |
| 33. | | Primary Care Group and the Positive Health Network had unsuccessful experiences with the developing of CE because they had not considered their vision, goals, or target groups for CE | | For successful CE it is important to transparently discuss shared goals, expectations and visions, and that both citizens and professionals feel the need for an equal partnership | The fact alone that organisations maintain all budgets creates a power imbalances, which is why the Positive Health Network is looking to develop a village budget |
| 34. | | The original aims/vision of the community-led initiative were overly ambitious | | No one could take this vision/aims seriously and therefore many previously engaged citizens dropped out of the initiative (they felt no connection to the vision/aims) | The new governance board therefore wants to develop an achieve and shared vision with underlying goals for the initiative moving forward |
| **Citizens and professionals had experienced a misalignment between citizens’ and professionals’ perspectives and motivations for CE** | | | | | |
| 35. | | At the community-led initiative, citizens and professionals have been involved | | Citizens and professionals had different goals and motivations which the citizens felt often clashed | This complicates the collaboration between citizens and professionals (as citizens and professionals tend to work on separate projects separately, rather than together) and leads to the fact that each has to make concessions |
| 36. | | - Volunteer work costs a lot of time and energy - The community-led initiative is in transition and is looking for new vision and ambition | | Because participant is older he has to protect his boundaries and he has noticed health and wellbeing is more important than ever | This has motivated him to set up a social club for people who want to talk and exchange tips about healthy ageing |
| 37. | | - Community-led initiative is in transition and is searching for which aims and activities should be continued | | Engaged citizens and professionals have differing goals and ambitions. Professionals are more outcome focussed and can therefore aim too high. Citizens meanwhile are engaged because of their own intrinsic motivations and because they want to increase their social connection within the community | These differences in aims should be openly discussed |
| 38. | | Both citizens and professionals were involved with the community-led initiative who different goals and interests | | The old governance board never ensured these differences in goals and interests were openly discussed and addressed because they were afraid it would make them vulnerable | That is why the new governance board has to ensure these differences in interests and goals are finally transparently discussed |
| 39. | | After the old governance board quit, the new board is doing some soul searching for a new vision and what the new options are | | Support worker fears that the initiative will start making the same mistakes if they keep organising the same practical/tangible activities without first developing a shared vision | - Support worker wants them to develop a clear vision and to organise activities around that - Support worker wants them to develop a collaboration with health care organisations and wants the initiative to be adopted within the local health care policy. - This shows a mismatch between citizens’ and support worker’s priorities |
| 40. | | With CE approaches, everyone (citizens, professionals, volunteers) involved has their own language, role, interests and scope | | Citizens often think and operate on a smaller (community-based) level. This is why professionals become irritated because they feel change is not happening fast enough | This shows that motivations between citizens and professionals are not aligned and that resource investments (esp. time and space) should be created to discuss these differences and address the motivations/interests of citizens. |
| 41. | | Community-led initiative was started by a local healthcare professional and a lot of like-minded residents got involved | | But because citizens’ and engaged professionals’ motivations were different (professionals were mostly motivated as they saw it as an opportunity to grow their client base), the dynamic of the initiative changed | Citizens no longer felt connected to the initiative due to different motivations and many stopped their engagement |
| 42. | | After the unsuccessful experience of the Policyholder Cooperation, the Board started looking for ways to better connect to the communities and align with citizens’ motivations | | But this community-based approach is difficult for organisations to accept as they have their own interests and motivations regarding CE and have their own targets and goals to hit | For successful CE, organisations need to discuss these priorities with citizens and find a way to align the different motivations |
| **Acknowledge and address power imbalances** | | | | | |
| 43. | | Municipality wanted to approach the redesign of the village square differently with more participation from citizens | | - Municipality started the new project wanting to see citizens and the municipality as equals. They felt that municipality and citizens had different but equal knowledge and skills - Municipality dared to step out of the traditional division of roles | Because of this new approach citizens were fully involved in the decision-making |
| 44. | | Health care system does not appreciate the value of the community-led initiative and also does not know what the initiative’s function or role could be within the wider system | | Professionals must learn that engaged citizens are equal partners and to appreciate that community-led initiatives can provide support to the wider community | Only then can a successful collaboration between communities and the system be developed |
| 45. | | Citizens and professionals have different perspectives, languages and scope | | If only one citizen joins a working group/board, then that citizen is only provided the space to provide input and for the professionals then to ignore that input as that one engaged citizen is not seen as equal | Such involvement is not true participation as professionals go back to ‘bsuiness as usual’ by ignoring that citizen’s input |
| 46. | | The Policyholder Cooperative board members were citizens but at the same time they were also highly educated and administrators in their own sectors which meant they were very interested in governance issues themselves as well | | This made the regional health and care governance board feel like the ‘average citizen’ was still not represented on the Cooperation which made them question the value of the Cooperation as a whole | This meant that the Cooperation and the health and care board were unaligned and both perceived like no successful activities/projects were being implemented |
| **Share decision-making control** | | | | | |
| 47. | | Municipality has started to become convinced of the importance of involving citizens earlier on in projects | | But many policymakers still have the tendency to shape and a project before even having had a talk with citizens, as they are hesitant to share control with citizens | As such the early involvement of citizens is often not a priority. |
| 48. | | The Positive Health Network is looking to see if it can transfer important health care budgets (from the health care insurer) to the village council | | However, different perspectives and interests are a significant barrier to this development. Citizens have a more holistic view of health and wellbeing which means that this budget should span the different funders of the health care system. This would require the funders (especially health insurers) to dare to let go of some control | As health insurers are not prepared to do so, the village council still does not have its own budget to develop positive health for the community |
